# Enhanced optical bistability in slotted photonic crystal structure for microwave frequency generation

**DOI:** 10.1038/s41598-025-20992-w

**Published:** 2025-11-11

**Authors:** Akash Kumar Pradhan, Chandra Prakash, Sambit Satpathy, Jibitesh Kumar Panda

**Affiliations:** 1 School of Electronics Engineering, Vellore Institute of Technology, Chennai, 600127 India; 2 Department of Electronics and Communication Engineering, National Institute of Technology, Kurukshetra, India; 3 Department of Computer Science and Engineering, Galgotias College of Engineering, Greater Noida, Uttar Pradesh 203201 India; 4 Department of Mechatronics, Manipal Institute of Technology, Manipal Academy of Higher Education, Manipal, Karnataka 576104 India

**Keywords:** Photonic crystal, Slot, Nanocavity, Optical bistability, Energy science and technology, Engineering, Optics and photonics, Applied optics, Optical materials and structures, Optical techniques

## Abstract

This study proposes a novel silicon nanocrystal SiNC/SiO${}_{2}$ embedded slotted photonic crystal nanocavity employing precise width modulation to explore optical bistability and self-pulsing behavior for microwave signal generation. The designed cavity achieves an ultra-high quality factor (Q) of $2.15 \times 10^6$ and a low modal volume of $0.18 \, \mu m^3$. Theoretical modeling incorporating Kerr nonlinearity, two-photon absorption, and free carrier effects is used to analyze the bistability response. Simulation results reveal a low threshold power of $2 \mu W$ for optical bistability under a − 20 pm detuning condition. Additionally, the cavity demonstrates microwave frequency generation through self-pulsing oscillations, with a fundamental mode and observable second harmonic at 21.34 GHz. Fabrication tolerance is also evaluated, showing that the design sustains performance with up to 9% randomness in hole radii, 12% in x-position, and 20% in z-position of air holes. These findings confirm the feasibility of the proposed structure for low-power, high-frequency integrated photonic applications.

## Introduction

In the realms of photonics and optoelectronics, the phenomenon of optical bistability plays a pivotal role and has numerous applications in the design of devices such as memory elements, modulators^1^, and optical switches^2^. In recent years, generating microwave frequencies through the interplay between optical bistability and self-pulsing has garnered significant attention^3^. Self-pulsing is possible only when the cavity resides on the high-energy branch of the bistability^4^. Optical bistability refers to a system exhibiting two distinct output values in response to a single input^5^. While researchers have demonstrated successful applications of bistability in photonic crystal fibers^6^ and optical fibers^7^, the latter poses compatibility challenges with the CMOS platform. As a solution, silicon-based optical devices have emerged as the preferred choice for implementing optical bistability in these contexts. In addition to being compatible with CMOS technology, silicon possesses attributes such as two-photon absorption (TPA), free carrier absorption (FCA), and free carrier dispersion (FCD). The essential nature of two-photon absorption (TPA), free carrier absorption (FCA), and free carrier dispersion (FCD) makes silicon a key material for enabling optical bistability and self-pulsing^8–10^. Consequently, silicon-based structures like ring resonators^11,12^, periodic dielectric waveguides^13^, and photonic crystal^14^ configurations are employed to induce bistability and self-pulsing^4^ phenomena. Notable advancements include the demonstration of optical bistability in a tunable gourd-shaped silicon ring resonator by Chen et al.^15^ and the exploration of thermo-optic bistability in high-index doped silica ring resonators by Hu et al.^16^ Among these, photonic crystal cavities^17–20^ have emerged as the preferred choice, primarily attributed to their high-quality factor (Q) and small modal volume (V). The selection is motivated by the advantageous high Q/V ratio, as the intensity of nonlinear interactions between light and the medium relies on this parameter. This enhanced nonlinear interaction facilitates the realization of optical devices with a compact footprint and low operating power. A principle utilized in recent designs such as the all-optical memory based on surface plasmons and Kerr-type nonlinear cavities proposed by Jafari and Danaie^21^ , and in bistable optical switches operating near exceptional points as studied by Zhang etal^22^. Almeida et al. effectively demonstrated bistability within a silicon-based ring resonator, achieving this outcome with a power expenditure of 800 $\upmu$W^10^. In contrast, Notomi et al. realized optical bistability in a photonic crystal cavity with a remarkably lower input power of 40 $\upmu$W. Recently, Li et al. demonstrated low-threshold bistability using a photonic crystal Fabry–Perot cavity based on a three-dimensional Dirac semimetal. Yang et al^23^. Further reported topologically protected bistability using a two-dimensional photonic crystal L6 nanocavity dimer array, which highlights the evolving versatility of photonic crystal platforms^24^. There is an additional need to reduce the threshold input power for bistability^17^.Moreover, when bistability phenomena are used for self-induced oscillation, the generated frequency is also an important performance parameter. In this context, silicon-based photonic crystal structures have been reported to generate microwave frequencies up to 10 GHz through self-induced oscillations^20^. More recently, Shetewy et al. demonstrated high-frequency self-pulsing oscillations in an active silicon micro-ring cavity, confirming the potential of engineered resonators to achieve GHz-range dynamics through nonlinear effects^25^. To further reduce the threshold power and extend the range of self-induced oscillation frequencies, researchers are exploring alternative silicon-compatible nonlinear materials with higher Kerr and TPA coefficients than silicon. Silicon nanocrystal(SiNC/SiO${}_{2}$) based nanomaterial recently attracted the attention of resaerchers due to its higher nonlinearity^26^. The Kerr and TPA coefficient of silicon nanocrystal is 100 and 10 times more than silicon^27^. The design limitations of SiNC/SiO${}_{2}$-based photonic crystals arise from the inherent challenge of achieving strong light confinement due to the refractive index of silicon being 2. To overcome this issue, a viable solution involves incorporating SiNC/SiO${}_{2}$ as slots within silicon-based photonic crystal structures, following a similar approach as demonstrated in previous studies^28–31^. This innovative configuration of SiNC/SiO${}_{2}$-based slotted photonic crystal structures proves effective in providing both temporal and spatial confinement of light.

With the goal of realizing bistability with a significantly low threshold power for potential applications in microwave frequency generator, this research centers on the design of a silicon nanocrystal embedded slotted photonic crystal nanocavity. The subsequent paragraph outlines the key aspects of this study.

The subsequent section introduces the nanocavity design, followed by an examination of mathematical modeling for bistability. Following this, the subsequent segment delves into simulation outcomes concerning bistability, while another section investigates the utilization of bistability for microwave frequency generation. The discussion on the effects of fabrication imperfections on the frequency response ensues thereafter. Ultimately, the research results are succinctly summarized in the concluding section.

## Design of the nanocavity

**Fig. 1:**
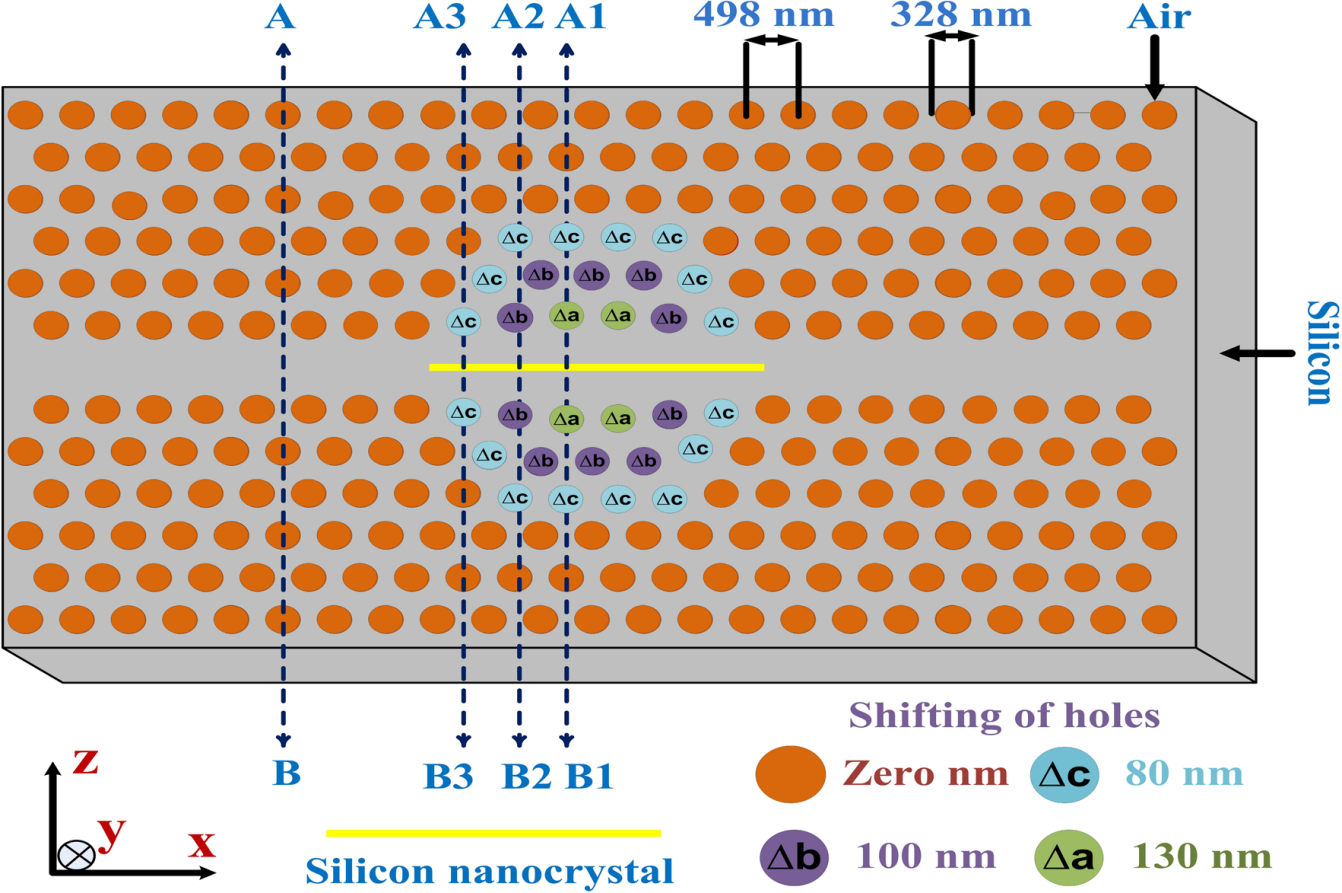
Design of the proposed SiNC/SiO${}_{2}$ embedded slotted photonic crystal cavity. The cavity is formed by shifting air holes along the z-direction by $\mathrm {\Delta }$a, $\mathrm {\Delta }$b, and $\mathrm {\Delta }$c to form the nanocavity. The holes labeled $\mathrm {\Delta }$a, $\mathrm {\Delta }$b, and $\mathrm {\Delta }$c are shifted from their original positions by 130 nm, 100 nm, and 80 nm, respectively. The slab thickness is considered to be 250 nm.

The design of the proposed SiNC/SiO${}_{2}$-based slotted photonic crystal nanocavity is depicted in Fig. 1. The photonic crystal structures were created by perforating holes in a silicon slab in a hexagonal pattern. Subsequently, a waveguide structure was formed by eliminating a single row of holes.

**Fig. 2:**
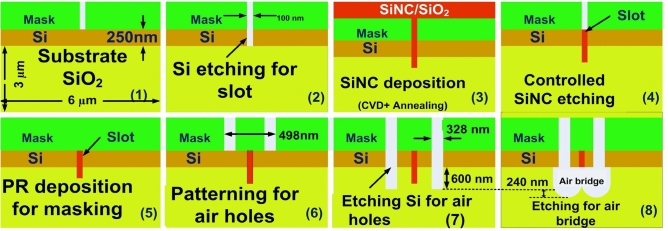
Fabrication required for the design of nanocavity as^32^.

The lattice constant *(a)* of the photonic crystal structure is 498 nm, and the radius of the holes is approximately 0.3 times the lattice constant, i.e., 164 nm. The thickness of the silicon slab is considered to be 250 nm. In Figure 1, the positions of the air holes have been adjusted in the z-direction to create a cavity, with some holes being shifted upward and others downward from their original position^33^. These adjustments are denoted by $\Delta a$, $\Delta b$, and $\Delta c$, which are the parameters defining the specific shifts. Specifically, the holes are moved in the z-direction by amounts of $\pm \Delta a$, $\pm \Delta b$, and $\pm \Delta c$. Here, the “+” sign indicates an upward shift of the air holes, while the “–” sign indicates a downward shift. These modifications are crucial for the cavity’s design and functionality. At the center of the cavity, a slotted region filled with (SiNC/SiO${}_{2}$) material is embedded to ensure strong optical confinement. This slot has a width of 100 nm and extends longitudinally along the waveguide axis. The slot height matches the silicon slab thickness, which is 250 nm, and the refractive index of the slot material is 2, corresponding to the effective index of (SiNC/SiO${}_{2}$) nanocomposite^28^. The slot is precisely etched and aligned between the shifted air holes to maximize the field confinement within the high-index nonlinear region. While the slotted photonic crystal proposed in this study has not undergone fabrication, it can be manufactured using a methodology similar to that outlined in^32,34–36^.

Furthermore, the specific fabrication process is illustrated in detail in Fig. 2. The fabrication begins with an SOI wafer, where SiO2 is 3 $\upmu$m thick, and Si is 250 nm thick. A mask (PR) is deposited, defining an 100 nm wide slot in step 1 of Fig. 1. Si is etched in step 2, followed by LPCVD deposition of Si and SiO2 in a 1:10 volumetric ratio^27^. High-temperature annealing forms (SiNC/SiO${}_{2}$), and controlled etching leaves (SiNC/SiO${}_{2}$) up to the Si layer’s top surface.In step 5, PR is deposited to mask the wafer, and air hole patterning follows. Deep Reactive Ion Etching (DRIE) creates air holes in Si, penetrating the (SiO${}_{2}$) substrate up to $\mathrm {\sim }$600 nm. In the final step, controlled wet etching removes $\mathrm {\sim }$240 nm of the underlying (SiO${}_{2}$). This process yields an air bridge SPCW structure, introducing a shallow cone beneath the SiNC/SiO2 slot, which does not significantly impact device characteristics as the electric field is primarily confined above this region. Unlike conventional photonic crystal cavities, the structure presented here integrates a high-index (SiNC/SiO${}_{2}$) slot at the cavity center, which significantly enhances nonlinear light-matter interactions and allows for bistability at a lower threshold power. This hybrid design, which combines width modulation and non-linear material enhancement, contributes to both spatial and temporal confinement of light, an advancement not widely explored in previous studies.

To evaluate the performance of the cavity, particularly its Q factor, the frequency response and Q factor were analyzed with various combinations of $\mathrm {\Delta }$a, $\mathrm {\Delta }$b and $\mathrm {\Delta }$c. This integrated approach allows for a comprehensive evaluation of the cavity’s characteristics and performance.

**Fig. 3:**
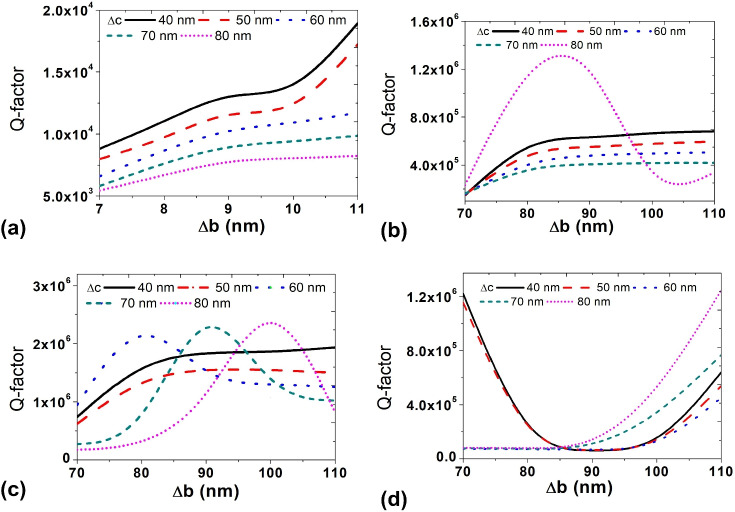
Variation of Q-factor as a function of $\mathrm {\Delta }$b and $\mathrm {\Delta }$c for different fixed values of $\mathrm {\Delta }$a: (**a**) $\mathrm {\Delta }$a = 150 nm, (**b**) $\mathrm {\Delta }$a = 140 nm, (**c**) $\mathrm {\Delta }$a = 130 nm, and (**d**) $\mathrm {\Delta }$a = 120 nm. These figures demonstrate the interplay between structural displacement parameters and their role in tuning the cavity’s Q-factor. Higher Q-factors are observed at specific combinations of $\mathrm {\Delta }$b and $\mathrm {\Delta }$c, revealing optimal configurations for light confinement and resonance.

**Fig. 4:**
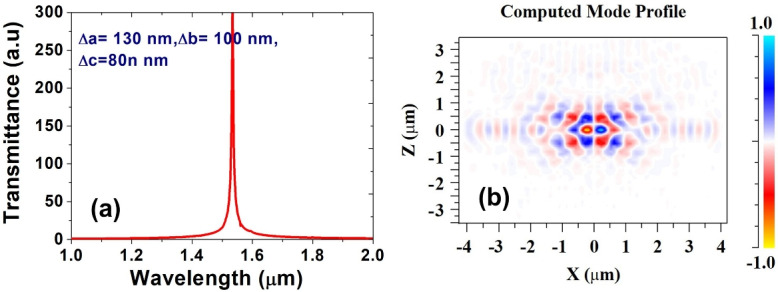
(**a**) Frequency response of nanocavity (**b**) Mode profile of the cavity at resonating mode.

### Optimization of cavity

The modulation of waveguide width plays a crucial role in the formation of cavities, influencing the characteristics of the resulting structures. In this context, precise adjustments to the positions of holes designated as $\mathrm {\Delta }$a, $\mathrm {\Delta }$b, and $\mathrm {\Delta }$c in the z-direction are essential for achieving high-Q factor cavities. A structured optimization methodology based on parametric sweeping was followed. Using 3D Finite-Difference Time-Domain (FDTD) simulations, the parameters $\mathrm {\Delta }$a, $\mathrm {\Delta }$b, and $\mathrm {\Delta }$c were varied in defined increments, and the corresponding Q-factors were analyzed to identify the configuration offering maximum cavity performance. This non-empirical approach allowed systematic convergence toward the optimal design. To initiate the simulation process, the value of $\mathrm {\Delta }$a has been set to 150 nm. A series of nanocavities has been meticulously designed by systematically varying the value of $\mathrm {\Delta }$b within the range of 70 nm to 110 nm and $\mathrm {\Delta }$c within the range of 40 nm to 80 nm. The simulation outcomes, represented in Fig. 3a, illustrate the variation in the Q-factor for these designed nanocavities.The figure depicts an increase in the Q-factor value as $\mathrm {\Delta }$b rises while maintaining a constant $\mathrm {\Delta }$c. Specifically, for $\mathrm {\Delta }$c values of 60 nm, 70 nm, and 80 nm, the Q-factor values exhibit an upward trend with increasing $\mathrm {\Delta }$b. However, it becomes evident that this trend starts to saturate within the range of 90 nm to 110 nm. Notably, when $\mathrm {\Delta }$c is set to 60 nm and 70 nm, the Q-factor values follow a consistent increasing pattern. In contrast, the Q-factor values decrease as $\mathrm {\Delta }$c increases while keeping $\mathrm {\Delta }$b constant. Figure 3b illustrates the variation of the Q-factor concerning changes in the values of both $\mathrm {\Delta }$b and $\mathrm {\Delta }$c, while maintaining a constant $\mathrm {\Delta }$a value of 140 nm. The depicted trend reveals an initial increase in Q-factor values with the rise in $\mathrm {\Delta }$b for constant $\mathrm {\Delta }$c values of 40 nm, 50 nm, 60 nm, and 70 nm. However, a distinctive pattern is observed when $\mathrm {\Delta }$c is set to 80 nm, where the Q-factor values increase with the ascending $\mathrm {\Delta }$b until reaching a peak at $\mathrm {\Delta }$b=90. Subsequently, a decreasing pattern ensues. Additional simulations were conducted for various combinations of $\mathrm {\Delta }$b and $\mathrm {\Delta }$c while maintaining a constant $\mathrm {\Delta }$a value of 130 nm. The corresponding Q-factor variations are presented in Fig.3c. The figure illustrates that, for $\mathrm {\Delta }$c values of 40 nm and 50 nm, the Q-factor values exhibit an increase followed by a saturation pattern as $\mathrm {\Delta }$b values increase. In contrast, for $\mathrm {\Delta }$c values of 60 nm, 70 nm, and 80 nm, the Q-factor values display an increasing pattern, reaching a peak value before decreasing. Notably, the curves corresponding to $\mathrm {\Delta }$c values of 60 nm, 70 nm, and 80 nm achieve their maximum Q-factor values at $\mathrm {\Delta }$b values of 80 nm, 90 nm, and 100 nm, respectively. Finally, the study involves the variation of the Q-factor with changes in the values of $\mathrm {\Delta }$b and $\mathrm {\Delta }$c, maintaining a constant $\mathrm {\Delta }$a value of 120 nm, as depicted in Fig. 3d. The figure reveals that the curves corresponding to $\mathrm {\Delta }$b values of 40 nm and 50 nm initially reach their peak values at $\mathrm {\Delta }$b=70 nm, gradually decreasing with an increase in $\mathrm {\Delta }$b, and then resume an upward trend around $\mathrm {\Delta }$b=100 nm. In contrast, the curves for $\mathrm {\Delta }$b values of 60 nm, 70 nm, and 80 nm exhibit very low Q-factors up to $\mathrm {\Delta }$b=90 nm, after which they start increasing again. Based on the data presented in Fig. 3, it can be inferred that a $\mathrm {\Delta }$a value of 150 nm results in a peak Q-factor of 1.8 $\mathrm {\times }$ ${10}^4$ when $\mathrm {\Delta }$b is set to 110 nm and $\mathrm {\Delta }$c is set to 40 nm. Similarly, for $\mathrm {\Delta }$a= 140 nm, the highest Q-factor of 1.23 $\mathrm {\times }$ ${10}^6$ is attained with $\mathrm {\Delta }$b= 85 nm and $\mathrm {\Delta }$c= 80 nm. Additionally, the combination of $\mathrm {\Delta }$a= 130 nm, $\mathrm {\Delta }$ b=100nm $\mathrm {\Delta }$ c=80nm yields a Q-factor value of 2.15 × 10^6^. This specific combination has been utilized in this study to investigate the optical bistability phenomena.

 In addition to the Q factor of the cavity, the transmission ratio is a crucial parameter that needs to be considered during the optimization process. The transmission ratio often has a trade-off relationship with the Q factor. While a higher Q factor signifies lower energy loss and sharper resonance, it can lead to a reduced transmission ratio, indicating less efficient coupling of light into and out of the cavity. 

It is important to clarify that in Fig. 3a–d the shifts a, $\mathrm {\Delta }$b and $\mathrm {\Delta }$c correspond to the displacement of the holes in the z-direction near the cavity region. These shifts were intentionally applied with asymmetric sign conventions: holes located above the W1 waveguide were shifted with positive values, and those below the waveguide were shifted with negative values. This asymmetry creates an “eye-like” opening around the cavity, improving optical confinement and leading to higher Q-factors. The resulting Q-factor curves are not symmetric with respect to simply changing the sign of these deltas, as reversing the shifts would yield a structurally different cavity with degraded confinement. Furthermore, the opposite sign convention was not considered in our optimization because it would reduce the effective cavity space in the z-direction—an important constraint, since a nanocrystal is to be embedded within the cavity. Reduced volume could obstruct the nanocrystal’s placement and affect coupling with the confined optical mode. Hence, we optimized $\mathrm {\Delta }$a , $\mathrm {\Delta }$b , and $\mathrm {\Delta }$c using positive values for holes above and negative values for holes below the waveguide. The simulation results shown in Fig. 3a–d reflect this optimized asymmetric configuration.

Conversely, optimizing for a higher transmission ratio may result in a lower Q factor, suggesting increased energy loss but more efficient light coupling. Balancing these two parameters is essential to achieve optimal cavity performance.

### Response of the cavity

Figure 4a displays the frequency response of the newly proposed slotted photonic crystal cavity, indicating resonance at a wavelength of 1530 nm. In order to assess the mode profile at this resonant wavelength, a 3D Finite-Difference Time-Domain (FDTD) simulation was conducted, and the associated electric field profile is presented in Fig. 4b. The figure illustrates that the resonant wavelength is highly confined within the cavity, facilitating strong interaction of light with the silicon nanocrystal material.

**Fig. 5:**
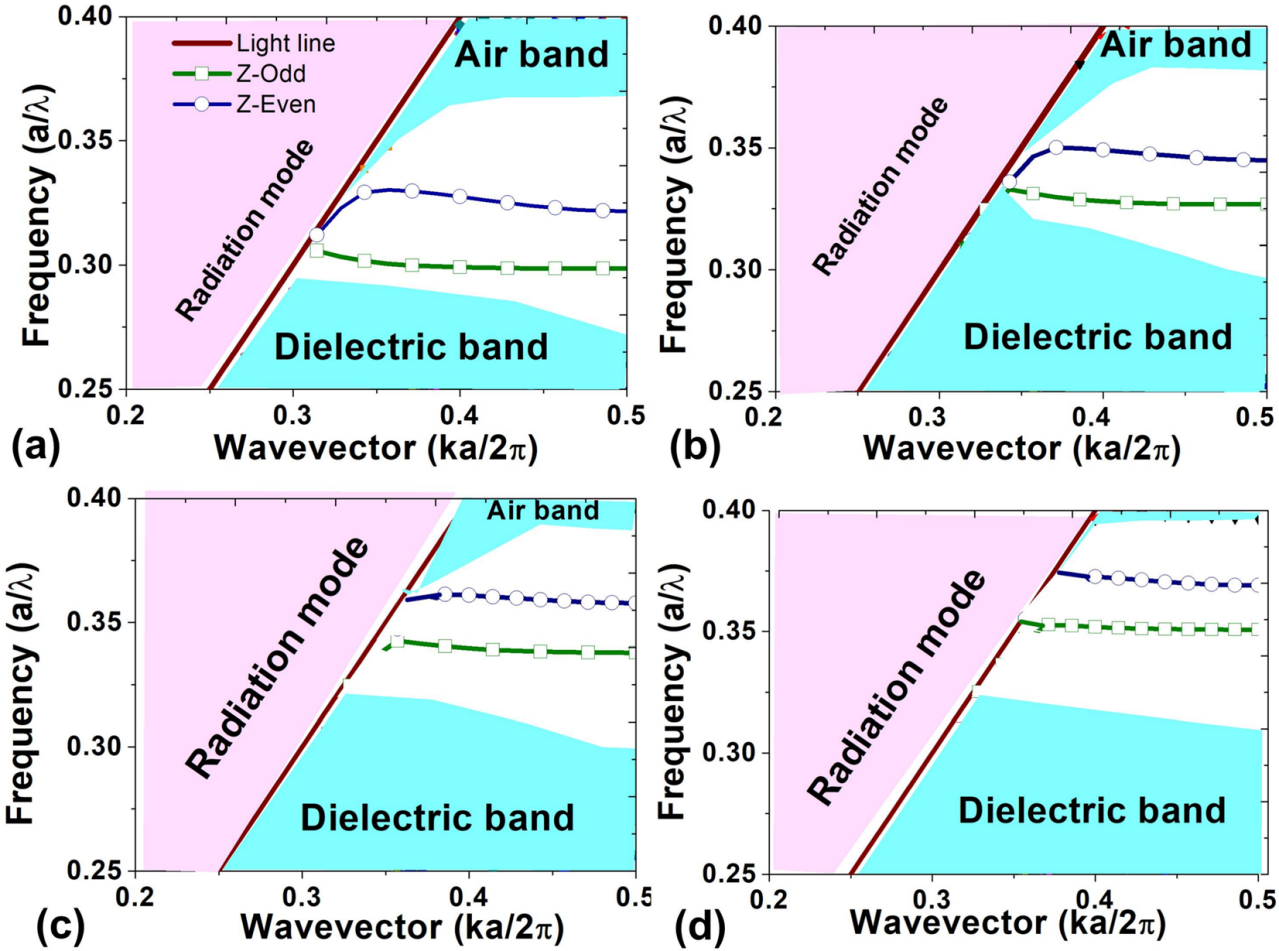
Dispersion diagram at different positions of nanocavity (**a**) AB (**b**) A1B1 (**c**) A2B2 (**d**) A3B3.

Additionally, the cavity’s response has been assessed using the plane wave expansion method^37^. In this regard, the dispersion diagram of the proposed structure has been analyzed in four different positions: AB, A1B1, A2B2, and A3B3, as illustrated in Fig. 1. In the AB position, where the slot is absent, it represents the waveguide section. The dispersion diagram for this position is depicted in Fig. 5a, revealing two guided bands referred to as Z-even and Z-odd bands. Notably, the resonating wavelength (1530 nm = *a*/$\lambda$ = 0.325) falls within the guided band, providing confirmation of the propagation of the resonant wavelength through the waveguide. The A3B3 and A2B2 positions represent the edges of the nanocavity, with A2B2 exhibiting a greater displacement in the z-positions of air holes compared to A3B3. The dispersion diagrams for the cavity at positions A1B1 and A2B2 are presented in Fig. 5b and c. The figures reveal that as the width of the waveguide decreases, the dispersion diagram shifts upward towards the air band. Consequently, the resonant wavelength falls within the bandgap and bandedge region. This shift results in the input light displaying a low group velocity at A3B3 and A2B2 positions. In other words, the modulation of width at these positions acts similarly to a mirror, influencing the propagation characteristics of the input light. The dispersion diagram for the central position (AB) of the cavity is depicted in Fig. 5d. The diagram indicates that the resonating wavelength is situated within the band, allowing the light to effectively interact with the slot material.

## Mathematical modelling of the slotted photonic crystal nanocavity

The temporal evaluation of light inside a photonic crystal nanocavity can be governed through the following equations, similar to those discussed in^38,39^ with some modifications, including the consideration of the Kerr effect.

1 $$\begin{aligned} \frac{dA}{dt}= & \frac{A}{2\tau } + i(\omega - \omega _o - \frac{\gamma _i^{kerr} |A|^2}{\hbar {\omega }})A + \sqrt{\frac{\sqrt{T_{\text {max}}} P_{\text {in}}}{2\tau }} - \frac{\gamma ^{TPA} |A|^2 A}{2\hbar {\omega }} - \frac{\gamma ^{FC} N_e A}{V}\end{aligned}$$

2 $$\begin{aligned} \frac{dN_{e}}{dt}= & -\frac{N_e}{\tau _{FC}} + \frac{\gamma ^{TPA} |A|^4}{2(\hbar {\omega })^2} \end{aligned}$$

Equation (1) describes the dynamics of the envelope *A* of the input light in an optical cavity, incorporating various nonlinear effects. The term $\frac{1}{\tau }=\frac{\omega }{Q}$ represents the linear loss of the cavity, while $\omega _{0}$ is the resonant frequency of the nanocavity. The term $\frac{1}{\tau }=\frac{\omega }{Q}$ represents the linear loss of the cavity, while $\omega$0 is the resonant frequency of the nanocavity. The phase change due to Kerr effect is denoted by $\gamma _i^{\text {kerr}} = \frac{(\omega _{o} c \hbar {\omega } n_{\text {Kerr}})}{(n_o^2 V)}$, where $n_{Kerr}$ is the Kerr nonlinear coefficient, and $n_0$ is the refractive index of the silicon nanocrystal^38,39^. Here, *A* represents the envelope of the input light with frequency $\omega$. The linear loss of the cavity is represented by $\frac{1}{\tau } = \frac{\omega }{Q}$. $\omega _o$ represents the resonant frequency of the nanocavity. So the frequency detuning factor is $\omega - \omega _o$. The change in phase due to the Kerr effect is contributed by the term $\gamma ^{kerr}_i = \frac{\omega _o c \hbar {\omega } n_{Kerr}}{n^2_o V}$. Where $n_{Kerr}$ and $n_0$ represent the Kerr nonlinear coefficient and refractive index of the silicon nanocrystal. Additionally, $\gamma ^{TPA} = \frac{c^2 \hbar {\omega } \beta ^{TPA}}{n^2_o V}$ represents losses due to two-photon absorption, with $\beta ^{TPA}$ as the TPA coefficient. The free carrier dispersion term is denoted by $\gamma ^{FC} = \gamma ^{FC}_{abs} + i\gamma ^{FC}_{dis} = \frac{c}{2n_o}(\sigma _{abs} - i\frac{2\omega _o}{c} \sigma _{dis})$, where $\sigma _{abs}$ and $\sigma _{dis}$ are the free carrier absorption and dispersion coefficients^38,39^. Eq. (2) governs the generation of free carriers ($N_e$) and involves a term $\tau _{FC}$ representing the free carrier lifetime. To study the bistability behavior, Eqs. (1) and (2) need to be linearized under steady-state conditions. Equation (1) can be decomposed into two real equations by expressing *A*(*t*) as the product of its magnitude $\left| A(t)\right|$ and phase $e^{i\Phi (t)}$. The derivative of the envelope *A* can then be expressed as follows:

3 $$\begin{aligned} \frac{dA}{dt} = \frac{d}{dt} (|A| e^{i\Phi }) = \left( \frac{d|A|}{dt} + i|A|\frac{d\Phi }{dt}\right) e^{i\Phi } \end{aligned}$$

Using Eq. 3, Eq. (1) can be rewritten as:

4 $$\begin{aligned} \frac{{d|A|}}{{dt}} + & i|A|\frac{{d\Phi }}{{dt}} = - \frac{{|A|}}{{2\tau }} + i\left( {\omega - \omega _{o} - \frac{{\gamma _{i}^{{kerr}} |A|^{2} }}{{\hbar \omega }}} \right)|A| \\ + & \sqrt {\left( {\sqrt {T_{{{\text{max}}}} } P_{{{\text{in}}}} } \right)/2\tau } e^{{ - i\Phi }} - \frac{{\gamma ^{{TPA}} |A|^{3} }}{{2\hbar \omega }} - \frac{{\gamma ^{{FC}} N_{e} |A|}}{v} \\ \end{aligned}$$

This yields two real equations for $\Phi$ and $\left| A\right|$:

5 $$\begin{aligned} \frac{d|A|}{dt} = -\frac{|A|}{2\tau } - \frac{\gamma _{\text {abs}}^{FC} |A| N_e}{V} - \frac{\gamma ^{TPA} |A|^3}{2\hbar \omega } + \cos \Phi \sqrt{\left( \sqrt{T_{\text {max}}} P_{\text {in}}\right) /2\tau } \end{aligned}$$

6 $$\begin{aligned} \frac{d\Phi }{dt} = (\omega - \omega _o - \frac{\gamma _{i}^{kerr} |A|^2}{\hbar \omega }) - \frac{\gamma _{\text {dis}}^{FC} N_e}{V} - \frac{\sin \Phi }{|A|}\sqrt{\left( \sqrt{T_{\text {max}}} P_{\text {in}}\right) /2\tau } \end{aligned}$$

For steady state condition $\frac{d\phi }{dt\ }=0$ , $\frac{d|A|}{dt}=0$ and $\frac{dN_e}{dt}=0$ . The free carrier value can be calculated as follow:

7 $$\begin{aligned} N_e = \frac{\tau _{FC} \gamma ^{TPA} |A_o|^4}{2(\hbar \omega )^2} \end{aligned}$$

By putting the value of $N_e$ in Eqs. (4) and (5) can be wriiten as follow:

8 $$\begin{aligned} \cos \Phi \sqrt{\left( \sqrt{T_{\text {max}}} P_{\text {in}}\right) /2\tau }= & \frac{|A|}{2\tau } + \frac{\gamma _{\text {abs}}^{FC} |A| N_e}{V} + \frac{\gamma ^{TPA} |A|^3}{2\hbar \omega }\end{aligned}$$

9 $$\begin{aligned} \sin \Phi \sqrt{\left( \sqrt{T_{\text {max}}} P_{\text {in}}\right) /2\tau }= & (\omega - \omega _o - \frac{\gamma _{i}^{kerr} |A|^2}{\hbar \omega })|A| - \frac{\gamma _{\text {dis}}^{FC} N_e |A|}{V}\end{aligned}$$

10 $$\begin{aligned} \tan \Phi = & \frac{{(\omega - \omega _{o} - \frac{{\gamma _{i}^{{kerr}} |A|^{2} }}{{\hbar \omega }}) - \frac{{\gamma _{{{\text{dis}}}}^{{FC}} (\tau _{{FC}} \gamma ^{{TPA}} |A|^{4} )}}{{2(\hbar \omega )^{2} }}/V}}{{\frac{1}{{2\tau }} + \frac{{\gamma _{r}^{{FC}} N_{e} }}{V} + \frac{{\gamma ^{{TPA}} |A|^{2} }}{{2\hbar \omega }}}} \\ = & \frac{{(\omega - \omega _{o} - \frac{{\gamma _{i}^{{kerr}} |A|^{2} }}{{\hbar \omega }}) - \frac{{\gamma _{{{\text{dis}}}}^{{FC}} (\tau _{{FC}} \gamma ^{{TPA}} |A|^{4} )}}{{2(\hbar \omega )^{2} }}/V}}{{\frac{1}{{2\tau }} + \frac{{\gamma _{r}^{{FC}} (\tau _{{FC}} \gamma ^{{TPA}} |A|^{4} )}}{{2(\hbar \omega )^{2} }}/V + \frac{{\gamma ^{{TPA}} |A|^{2} }}{{2\hbar \omega }}}} \\ \end{aligned}$$

By combining Eqs. (7–9), ${\left| A\right| }^2$ can be written as five degree polynomial as follow:

$$\begin{aligned} \left[ \left( \omega - \omega _o - \frac{(\gamma _i^{\text {kerr}} |A|^2)}{(\hbar \omega )} \right) |A| - \frac{(\gamma _i^{\text {FC}})}{V} \left( \frac{(\tau _{\text {FC}} \gamma ^{\text {TPA}} |A|^4)}{2(\hbar \omega )^2} \right) |A| \right] ^2 + \left[ \frac{|A|}{2\tau } + \frac{(\gamma _r^{\text {FC}} |A|)}{V} \left( \frac{(\tau _{\text {FC}} \gamma ^{\text {TPA}} |A|^4)}{2\hbar \omega } \right) + \frac{(\gamma ^{\text {TPA}} |A|^3)}{2\hbar \omega } \right] ^2 \end{aligned}$$

11 $$\begin{aligned} - \left( \sqrt{T_{\text {max}}} P_{\text {in}} \right) \frac{1}{2\tau } = 0 \end{aligned}$$

By solving Eq. (11) bistability behaviour can be calculated for *A*. The output power can be written as follow:

12 $$\begin{aligned} P_{\text {out}} = \frac{|A|^2}{\tau } \end{aligned}$$

## Result and discussion

**Fig. 6:**
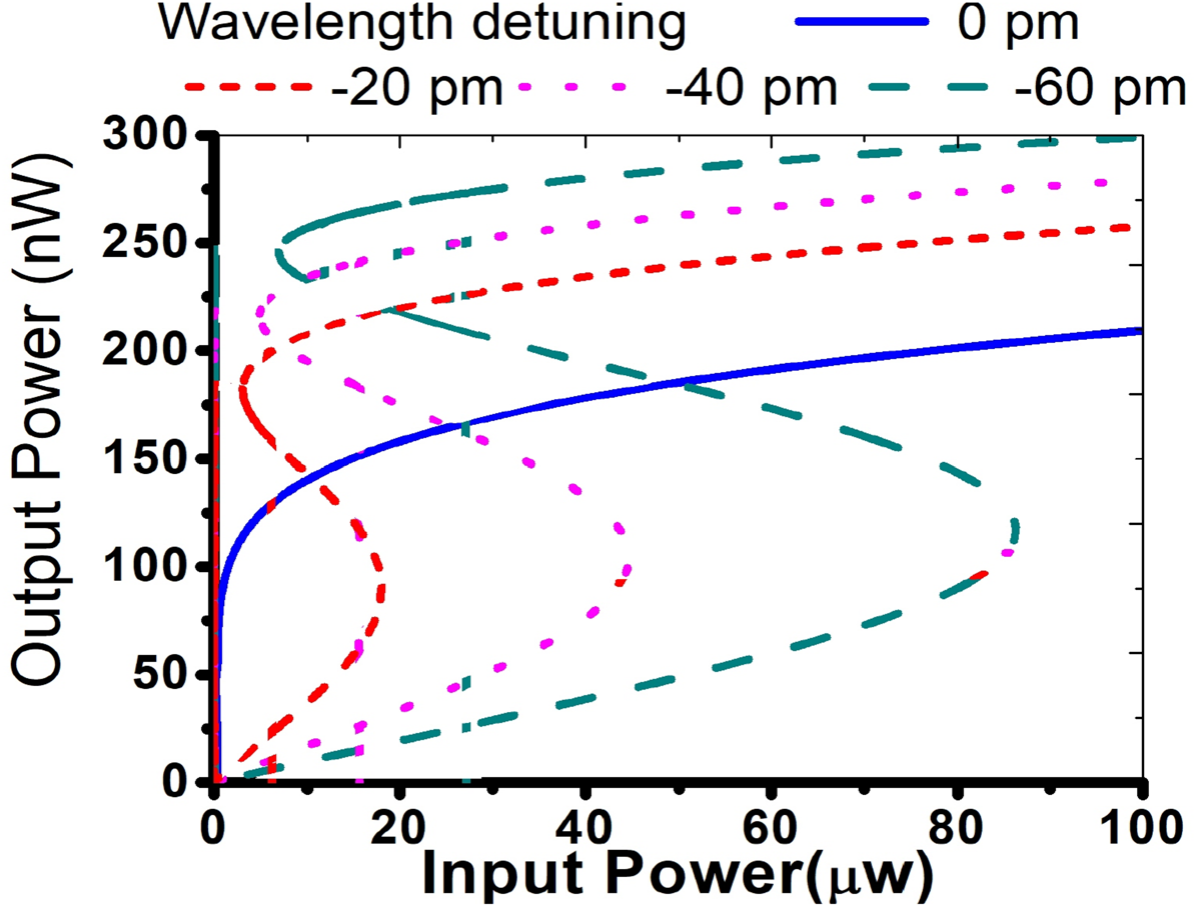
Transfer characteristics for different wavelength detuning.

The transfer characteristics of the nanocavity for various wavelength tunings have been computed utilizing Eqs. (10) and (11), as illustrated in Fig. 6. The simulation parameters essential for this study have been sourced from relevant literature, while additional parameters obtained from the Finite-Difference Time-Domain (FDTD) method are provided in Table 1. All information concerning silicon nanocrystals has been extracted from previously published papers.

It can be seen from Fig. 6 that there is no bistability at zero wavelength detuning, whereas a bistability phenomenon is observed for a detuning of − 20 pm. Furthermore, an increase in detuning corresponds to an elevation in the threshold power for bistability. The threshold power for optical bistability in this cavity is notably low at 2 $\upmu$W. This low threshold power is attributed to the combination of high nonlinear coefficients, a high Q-factor, and a low modal volume in the slotted photonic crystal cavity. To the best of the authors’ knowledge, such a low threshold power for Kerr- induced optical bistability has not been reported before. Moreover, to facilitate a clear understanding for the readers, a comparison table has been included in Table 2. This table highlights the key differences in threshold power between the thermo-optic bistability reported in the referenced study and the Kerr nonlinearity-induced optical bistability.

**Table 1:** Parameters required for the simulations.

Parameter	Symbol	Value	Source
Refractive index of silicon nanocrystal	*n*	1.99	^26^
TPA coefficient	$\beta ^\text {TPA}$	$50\times 10^{-11} \, \text {m/W}$	^28^
Kerr coefficient	$n_\text {Kerr}$	$4 \times 10^{-17} \, \text {m}^2/\text {W}$	^29^
Free-carrier lifetime	$\tau _\text {fc}$	$0.1\times 10^{-9} \, \text {s}$	^28^
Absorption coefficient	$\sigma _\text {abs}$	$14\times 10^{-22} \, \text {m}^2$	^29^
Resonant wavelength	$\omega$	$1530 \, \text {nm}$	FDTD
Quality factor	*Q*	$2.1\times 10^6 \,$	FDTD
Modal volume	*V*	$0.2\times 10^{-18} \, \text {m}^3$	FDTD

**Table 2:** Comparison of optical bistability reported in literature.

References	Platform	Threshold power	Q factor	Mechanism	Validation method
^40^	2D Photonic crystal (Rods in air)	Few mW	557	Kerr nonlinearity	Simulation
^14^	2D Photonic crystal (Holes in slab)	0.4 mW	7640	Kerr nonlinearity	Experimental
^41^	2D Photonic crystal (InGaAs)	37 $\upmu$W	2700	Kerr nonlinearity	Experimental
^42^	2D Photonic crystal (InGaAs)	4.2 mW	$1.8\times 10^5$	Kerr nonlinearity	Experimental
^17^	2D Photonic crystal (Holes in slab)	25 $\upmu$W	33400	Kerr + Thermo-optic	Experimental
^43^	1D Photonic crystal (Holes in slab)	1.6 $\upmu$W	$1\times 10^5$	Kerr + Thermo-optic	Experimental
^15^	Gourd shaped Silicon ring	3 mW	$2.2\times 10^5$	Kerr nonlinearity	Experimental
^16^	Highly doped silica ring	4–6 mW	NA	Thermo-optic	Experimental
^22^	Plasmonic racetrack resonator	2.2 mW/cm	NA	Kerr nonlinearity	Simulation
^24^	Photonic crystal dimer array	10 mW/m	NA	Topological feature	Simulation
This work	Slotted photonic crystal	2 $\upmu$W	$2.1\times 10^6$	Kerr nonlinearity	Simulation

## Application of bistability for microwave signal generation

Photonic crystal cavities play a pivotal role in integrated photonics owing to their high-quality factor (Q) and small modal volume (V). The effectiveness of nonlinear light-medium interactions is influenced by the high Q/V ratio, making these cavities valuable for realizing optical devices with compact footprints and low operating power. Various research groups have explored and demonstrated nonlinear optical phenomena such as stimulated Raman scattering, four-wave mixing, third harmonic generation, and optical bistability within photonic crystal cavities. The enhanced nonlinear interactions contribute to the development of optical devices with improved performance.

**Fig. 7:**
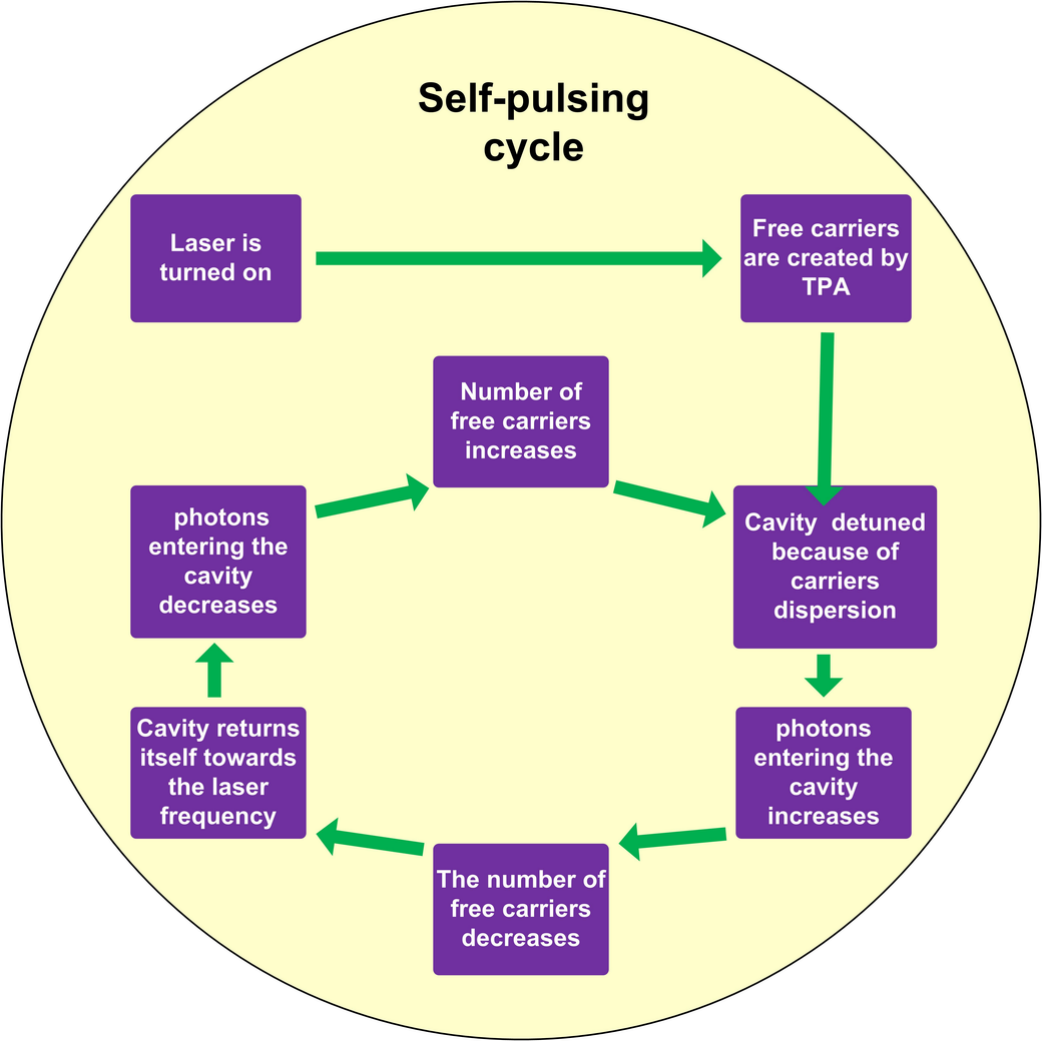
Schematic of self-pulsing mechanism inside the nanocavity.

Among the applications of nonlinear photonics, the optical generation of microwave signals stands out as an attractive prospect. Self-induced oscillations, resulting from optical bistability, have been a subject of exploration in the last few decades. For instance, Cazier et al. experimentally reported the generation of microwave signals in photonic crystal nanocavities^4,20,38^. Building on this line of research, this work also investigates the generation of microwave signals in slotted photonic crystal cavities, further expanding the understanding and potential applications of nonlinear photonics. The generation of microwave signal in nanocavity is due to the self pulsing mechanism as shown in Fig. 7^4,20,38^.

When the laser is activated, it induces the generation of free carriers within the cavity through two-photon absorption. This process leads to a detuning of the cavity resonance frequency relative to the laser frequency due to free-carrier dispersion. Consequently, the cavity’s transmission decreases, reducing the stored energy and, consequently, the number of free carriers. The cavity then self-adjusts, retuning towards the laser frequency, resulting in an increase in energy and the number of free carriers.

The analysis will be statrtd with linarizing Eqs. (5–7) in steady state condition for small variation in $(\delta A,\delta \varphi ,\delta N_e)$. The linear system can be written in matrix form as follows^38^:

13 $$\begin{aligned} \frac{d}{dt}\left( \begin{array}{c} \delta \left| A\right| \\ \delta \varphi \\ \delta N_e \end{array} \right) =M\left( \begin{array}{c} \delta \left| A\right| \\ \delta \varphi \\ \delta N_e \end{array} \right) \end{aligned}$$

Where the matrix M is-

$$M = \begin{pmatrix} -\frac{1}{2\tau } - \frac{{\gamma }^{FC}_{abs}N_e}{V} - \frac{3{\gamma }^{TPA}{\left| A\right| }^2}{2\hbar \omega } & -\sin \Phi \sqrt{\frac{\sqrt{T_{\text {max}}}P_{\text {in}}}{2\tau }} & -\frac{{\gamma }^{FC}_{abs}\left| A\right| }{V} \\[8pt] \frac{\sin \Phi }{{\left| A\right| }^2}\sqrt{\frac{\sqrt{T_{\text {max}}}P_{\text {in}}}{2\tau }} & -\frac{\cos \Phi }{\left| A\right| }\sqrt{\frac{\sqrt{T_{\text {max}}}P_{\text {in}}}{2\tau }} & -\frac{{\gamma }^{FC}_{dis}}{V} \\[8pt] \frac{2{\gamma }^{TPA}{\left| A\right| }^3}{{\left( \hbar \omega \right) }^2} & 0 & -\frac{1}{{\tau }_{FC}} \end{pmatrix}$$

since

$$\begin{aligned} \tan \Phi = & \frac{{\left( {\omega - \omega _{o} - \frac{{\gamma _{i}^{{kerr}} \left| A \right|^{2} }}{{\hbar \omega }}} \right) - \frac{{\gamma _{{dis}}^{{FC}} N_{e} }}{{V_{{eff}}^{{FC}} }}}}{{\frac{1}{{2\tau }} + \frac{{\gamma _{r}^{{FC}} N_{e} }}{V} + \frac{{\gamma ^{{TPA}} \left| A \right|^{2} }}{{2\hbar \omega }}}} \\ = & \frac{{\left( {\omega - \omega _{o} - \frac{{\gamma _{i}^{{kerr}} \left| A \right|^{2} }}{{\hbar \omega }}} \right) - \frac{{\gamma _{{dis}}^{{FC}} \frac{{\tau _{{FC}} \gamma ^{{TPA}} \left| A \right|^{4} }}{{2(\hbar \omega )^{2} }}}}{V}}}{{\frac{1}{{2\tau }} + \frac{{\gamma _{r}^{{FC}} \frac{{\tau _{{FC}} \gamma ^{{TPA}} \left| A \right|^{4} }}{{2(\hbar \omega )^{2} }}}}{V} + \frac{{\gamma ^{{TPA}} \left| A \right|^{2} }}{{2\hbar \omega }}}} = \frac{{\gamma _{{dis}}^{{FC}} }}{{\gamma _{{abs}}^{{FC}} }} \\ \end{aligned}$$

The free carrier dispersion coefficient $\left( {\gamma }^{FC}_{dis}\right)$ is much higher than the free carrier absorption coefficient (${\gamma }^{FC}_{abs}$).which gives $\tan \Phi \sim \infty$ and $\Phi \sim \frac{\pi }{2}$. Moreover, by neglecting the effect of TPA and free carrier absorption, the matrix M can be written as:

$$M \sim \left( -\frac{1}{{\left| A\right| }^2} \begin{array}{ccc} 0 & \sqrt{\frac{\sqrt{T_{\text {max}}}P_{\text {in}}}{2\tau }} & 0 \\ \sqrt{\frac{\sqrt{T_{\text {max}}}P_{\text {in}}}{2\tau }} & 0 & -\frac{{\gamma }^{FC}_{dis}}{V} \\ 0 & 0 & -\frac{1}{{\tau }_{FC}} \end{array} \right)$$

From the ‘Cayley-Hamilton theorem’s characteristics, the roots of the polynomial equation are found by solving the eigenvalues of the matrix M^38^.

14 $$\begin{aligned} P(X) = \det (XI - M) = (X + \frac{1}{{\tau }_{FC}})(X^2 + \frac{1}{{|A_0|^2}} \sqrt{\frac{\sqrt{T_{\text {max}}} P_{\text {in}}}{2\tau }}) \end{aligned}$$

By solving Eq. (14) the oscillation frequency can be calculated as^38^:

15 $$\begin{aligned} \Omega = \frac{1}{|A|} \sqrt{\frac{\sqrt{T_{\text {max}}} P_{\text {in}}}{2\tau }} \end{aligned}$$

**Fig. 8:**
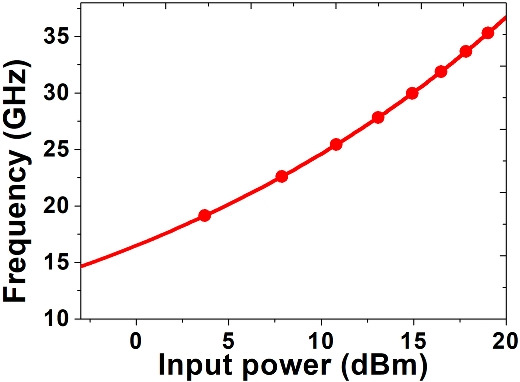
Variation of frequency with input power due to self-pulsing phenomena.

Although Eqs. (5) and (6) describe the steady-state behavior, to analyze transient dynamics, we consider small perturbations around the steady-state as shown in Eq. (13). The resulting system matrix M captures how fluctuations in field amplitude, phase, and carrier density evolve over time. By examining the eigenvalues of M, we can predict whether the system exhibits damped or growing oscillations—offering a complete picture of both steady-state and transient regimes. Now by using the Eq. (14), the variation of oscillation frequency with input power for − 20 pm detuning has been shown in Fig. 8.

**Fig. 9:**
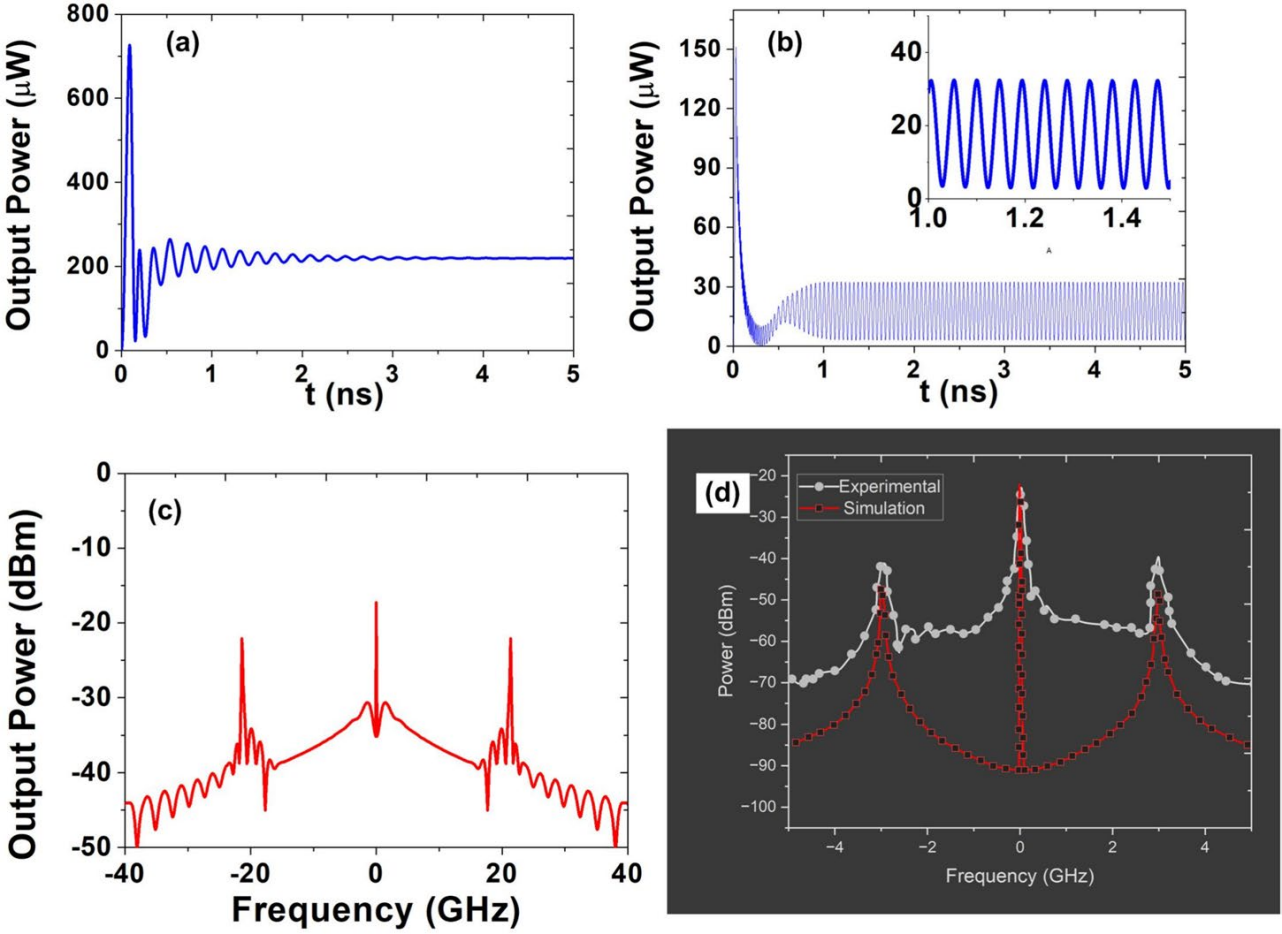
(**a**) The oscillation inside the cavity for an input power of 5 mW with frequency detuning 0 pm (**b**) The oscillation inside the cavity for an input power of 5 mW with frequency detuning − 20 pm (**c**) Frequency spectrum of the generated signal inside cavity. (**d**) Simulation validation with previously reported literature^20^.

Moreover, the amplitude and frequency spectrum of the output microwave signal can be calculated by numerically solving Eqs. (1) and (2)^4,20^. The output microwave signal power has been calculated for an input power of 5 mW and frequency detuning of 0 pm and − 20 pm. The corresponding amplitude spectra are shown in Fig. 9a and b. As illustrated in the figure, for a detuning of 0 pm, the oscillation dies out, whereas for − 20 pm, the output shows nonattenuated oscillation. Additionally, the spectrum of the generated microwave frequency for 5 mW input power and − 20 pm frequency detuning is shown in Fig. 9c. From Fig. 9c, it is evident that the fundamental oscillation occurs at around 0 GHz, while the second harmonic is at approximately 21.34 GHz. Additionally, the amplitude of the second harmonic is 15 dBm below that of the fundamental, confirming the presence of sinusoidal oscillation. Furthermore, to validate our simulation model, we have included a comparison with a previously reported experimental result^20^ and it is presented in Fig. reff9(d). As is evident from the figures, there is good agreement between the simulation and the experimental results, which lends additional credibility to our approach.

## Effect of fabrication imperfection on cavity performances

**Fig. 10:**
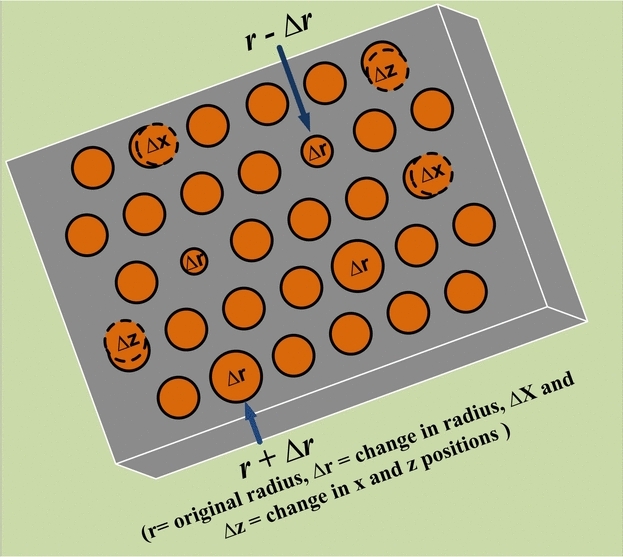
Possible fabrication imperfections occurred during fabrication of nanocavity.

Further examinations have been conducted to ascertain the device’s operational sustainability in the face of diverse structural imperfections that may emerge during the fabrication of nanocavity, building upon the previously calculated frequency response. In the course of these investigations, the frequency response and Q-factors have been analyzed to accommodate random variations in the radii, x and z-positions of the air holes, as illustrated in Fig. 10.

**Fig. 11:**
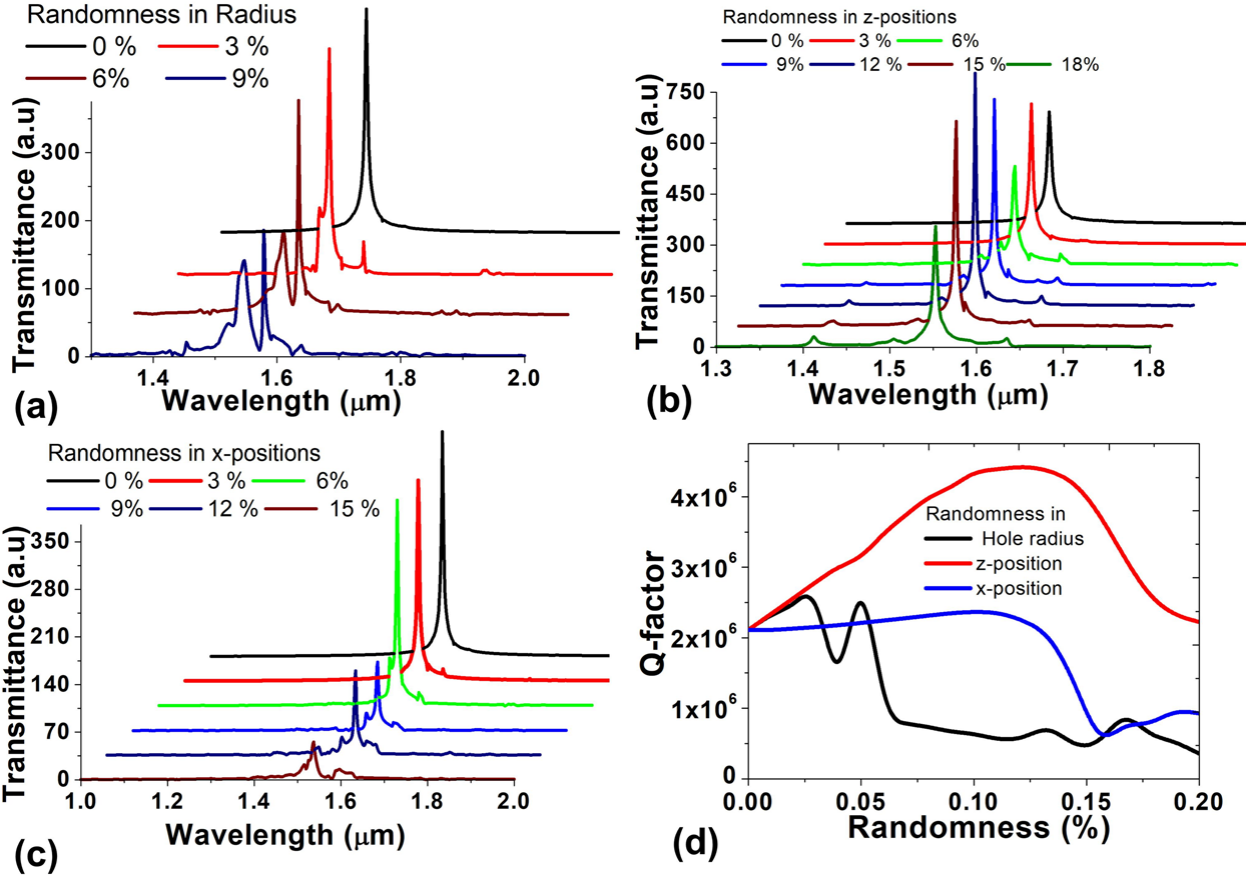
Effect on the frequency response of nanocavity due to randomness in (**a**) hole radii (**b**) z-positions (**c**) x-positions (**d**) variation of Q-factor for all randomness.

In the initial phase, the radii of the holes undergo random variations within a range of 0% to 9%, relative to the previously considered radius. This stochastic variation is expected to impact the resonance frequency and Q-factor of the cavity, with the results documented and illustrated in Fig. 11a and d. The figure indicates that up to a 6% change in hole radius, the transmission spectrum remains relatively stable. Notably, there is no significant deviation, and the quality factor does not decrease below 70% of the ideal Q-factor value, which is 2.15 $\mathrm {\times }$ ${10}^6$. However, beyond 9% randomness, there is a decrease in the Q-factor due to the emergence of new resonant wavelengths and a reduction in the linewidth of the original resonant wavelength, which is at 1533 nm. Hence, it can be inferred that the device exhibits tolerance up to 6% in random variations in hole radii. Additional simulations have been conducted to assess the cavity’s performance under variations in the in-plane x and z-positions. Initially, the z-positions of the holes were randomly adjusted within a range of 0–20%, and the corresponding variations in Q-factor were plotted in Fig. 11d. Interestingly, an observation emerged indicating an increase in Q-factor with the augmentation of randomness. This phenomenon is attributed to the adjustment of values for $\mathrm {\Delta }$a, $\mathrm {\Delta }$b, and $\mathrm {\Delta }$c as the randomness in z-position increases. This adjustment results in a narrower resonant spectrum, as illustrated in Fig. 11b. It is noteworthy that the cavity design in this study relies on the manipulation of the z-position of the holes. Consequently, introducing randomness in the z-position appears to yield a more favourable combination of $\mathrm {\Delta }$a, $\mathrm {\Delta }$b, and $\mathrm {\Delta }$c, contributing to an enhancement in the overall Q-factor. The Q-factor of the cavity can uphold the ideal Q-factor even with a randomness in the z-position of up to 20%. In the final set of simulations, the x-position of the holes was randomly adjusted within a range of 0–20% from their ideal positions. The corresponding outcomes, illustrating the resonant spectrum and the variation in Q-factor, are presented in Fig. 11c and d, respectively. Fig. 10d reveals that the Q-factor remains relatively constant for randomness levels up to 12%. Beyond this threshold, a subsequent decrease in the Q-factor is observed. Notably, at 15% randomness, there is a significant alteration in the transmission spectrum. Importantly, it can be inferred that the cavity’s transmission spectrum is not significantly affected by variations in the x-position, and the proposed cavity design demonstrates a tolerance of up to 12% random variation in the x-position. In summary, considering the cumulative impact of variations, the proposed nanocavity exhibits greater sensitivity to randomness in hole radii compared to in-plane positions. Furthermore, within the in-plane positions, it is observed that the cavity is more responsive to variations in the x-position than the z-position.

## Conclusion

In conclusion, a novel silicon nanocrystal embedded slotted photonic crystal cavity has been designed by modulating width of a photonic crystal waveguide to explore bistability phenomena. High Q-factor of 2.15 $\mathrm {\times }$ ${10}^6$ and small modal volume of 0.18 ${\upmu }\text {m}^3$ have been achieved for the proposed nanocavity, showcasing its effectiveness. The dispersion diagram at different positions of the nanocavity has been calculated using the plane wave expansion method, providing insight into the physical mechanism behind the cavity design through width modulation. Mathematical modeling has been employed to analyze the bistability phenomena, incorporating all nonlinear effects such as Kerr nonlinearity, two-photon absorption (TPA), four-wave mixing (FCA), and free carrier dispersion effects. The bistability characteristics of the proposed nanocavity have been calculated for various wavelength tunings, including 0 pm, − 20 pm, − 40 pm, and − 60 pm. The smallest threshold power observed for bistability was determined to be 2 $\upmu$W for a detuning of − 20 pm. Furthermore, an analysis of the self-pulsing phenomena of the nanocavity has been conducted to investigate its capability for optically generating microwave frequencies. Simulation results indicate that the slotted cavity can generate frequencies in the gigahertz range, attributed to the interaction between two-photon absorption and free carrier dispersion. Additionally, the fabrication tolerance of the proposed nanocavity has been assessed by examining its frequency response under different levels of randomness in hole radii and in-plane positions. The results demonstrate that the proposed design can tolerate randomness levels of 9%, 12%, and 20% in hole radii, x-position, and z-position, respectively.

## Data Availability

The datasets used and/or analysed during the current study available from the corresponding author on reasonable request.
